# Are community-based nurse-led self-management support interventions effective in chronic patients? Results of a systematic review and meta-analysis

**DOI:** 10.1371/journal.pone.0173617

**Published:** 2017-03-10

**Authors:** Azzurra Massimi, Corrado De Vito, Ilaria Brufola, Alice Corsaro, Carolina Marzuillo, Giuseppe Migliara, Maria Luisa Rega, Walter Ricciardi, Paolo Villari, Gianfranco Damiani

**Affiliations:** 1 Department of Public Health and Infectious Diseases, Sapienza University of Rome, Rome, Italy; 2 School of Nursing, Università Cattolica del Sacro Cuore, Rome, Italy; 3 Institute of Hygiene, Università Cattolica del Sacro Cuore, Rome, Italy; Helsingin Yliopisto, FINLAND

## Abstract

The expansion of primary care and community-based service delivery systems is intended to meet emerging needs, reduce the costs of hospital-based ambulatory care and prevent avoidable hospital use by the provision of more appropriate care. Great emphasis has been placed on the role of self-management in the complex process of care of patient with long-term conditions. Several studies have determined that nurses, among the health professionals, are more recommended to promote health and deliver preventive programs within the primary care context. The aim of this systematic review and meta-analysis is to assess the efficacy of the nurse-led self-management support versus usual care evaluating patient outcomes in chronic care community programs. Systematic review was carried out in MEDLINE, CINAHL, Scopus and Web of Science including RCTs of nurse-led self-management support interventions performed to improve observer reported outcomes (OROs) and patients reported outcomes (PROs), with any method of communication exchange or education in a community setting on patients >18 years of age with a diagnosis of chronic diseases or multi-morbidity. Of the 7,279 papers initially retrieved, 29 met the inclusion criteria. Meta-analyses on systolic (SBP) and diastolic (DBP) blood pressure reduction (10 studies—3,881 patients) and HbA1c reduction (7 studies—2,669 patients) were carried-out. The pooled MD were: SBP -3.04 (95% CI -5.01—-1.06), DBP -1.42 (95% CI -1.42—-0.49) and HbA1c -0.15 (95% CI -0.32–0.01) in favor of the experimental groups. Meta-analyses of subgroups showed, among others, a statistically significant effect if the interventions were delivered to patients with diabetes (SBP) or CVD (DBP), if the nurses were specifically trained, if the studies had a sample size higher than 200 patients and if the allocation concealment was not clearly defined. Effects on other OROs and PROs as well as quality of life remain inconclusive.

## Introduction

The global burden of non-communicable diseases (NCDs) is increasing rapidly and is expected to reach a prevalence of 57% in 2020, when such chronic conditions will outnumber acute conditions [1] and are likely to kill 38 million people each year [2]. In addition, over the next 20 years, NCDs are projected to cost more than US$ 30 trillion to the health systems, with a dramatic impact on productivity and quality of life [3]. The growing prevalence of NCDs, the aging population, rising patient expectations and the pressing need to contain costs lead to an increasing demand for primary care services, long term care services and reforms that move care from hospitals to the community, providing both first contact care and continuing care of individuals [4,5]. According to the Medical Home Model, the Institute for Healthcare Improvement (IHI) Model and the Chronic Care Model, only a productive interaction between an informed, activated patient and a prepared, proactive practice team can lead to improved outcomes [6]. The caregiver team must be patient-centered, coordinated, multidisciplinary, multi-professional and skilled in self-management support [7,8].

In this health care context, the transfer of tasks from medical doctors to appropriately trained nurses (so-called ‘task shifting’) can reduce both the workload of physicians and the direct cost of care, while achieving the same high quality of care, good health outcomes and, eventually, higher levels of patient satisfaction [4, 9, 10]. The effectiveness of task shifting in primary care, together with changing the skill mix, has been well reported in the literature [11–13] and is gaining growing acceptance among policy-makers [14]. Thus, the WHO has recommended that “continuous monitoring and evaluation must therefore be established as an integral component of the implementation process for task shifting […] and operational research should be developed alongside this implementation process” [15]. Moreover, nurses are already recognized as playing increasingly important roles in primary health care, especially in long-term care programs and in discharge planning programs for in-patients with chronic diseases [16–18].

Primary care must regain its central role in the frontline management of chronic diseases, because poor control at this level leads inexorably to hospital overcrowding due to the need to treat complications [19, 20]. To achieve this, great emphasis has been placed on the role of patient self-management, underlining its importance in primary care [8] and in the complex process of the care of patients with long-term conditions [21, 22]. Nurses, because of their traditional holistic perspective, are well versed in self-care support and must play a leading role in the administration of these systematic educational interventions focused on preserving or enhancing health and self-management goal achievement of a patient previously clinically assessed with a chronic disease. Self-monitoring (of symptoms or of physiologic processes) and decision making (managing the disease treatment or exacerbation or its impact through self-monitoring) are the aims of the interventions [23]. There are several primary studies that compare the impact of nurse-led interventions to support patient self-management with the more usual care-in-the-community programs for chronic patients [24–26]. However, to our knowledge, no systematic reviews on this specific topic are available in the literature; we therefore aim to provide such a systematic review in this study, and we also try to identify specific characteristics that might make interventions more effective.

## Materials and methods

### Selection criteria and search strategy

We carried out a systematic review of randomized control trials (RCTs) of nurse-led self-management support interventions performed with any method of communication exchange or education in a community setting on patients >18 years old with a diagnosis of chronic disease or multiple morbidity (see Table 1 for definitions). For this purpose, we drafted a protocol based on the population, intervention, comparison and outcome (PICO) approach [27] and the recommended guidelines for the reporting of systematic reviews and meta-analyses [28].

**Table 1:** Definitions of setting and interventions.

*Setting/Intervention*	*Definition*
**Nurse-led Self-Management Support intervention**	A systematic educational intervention that was targeted toward patients previously clinically assessed with a chronic disease. Nurse assessed determinant to provide a tailored educational intervention through an holistic perspective, focused on preserving or enhancing health and patient’s self-management goal achievement. Nurse provided health education to promote compliance and a healthy lifestyle. The intervention is finalized to help patient actively participate in either or both of the following: self-monitoring (of symptoms or of physiologic processes) or decision making (managing the disease treatment or exacerbation or its impact through self-monitoring).The intervention could be carried out by face to face encounters or consultation followed by telephone follow up. All telephone calls including prescriptions and patient concerns were addressed by the nurse who facilitated consultation with physician or other health professionals, if necessary.
**Usual Care**	Participants assigned to the usual medical care (control) group continued on-going care from their medical primary care provider (General Practitioner, Primary Care Physician) without any structured educational intervention.
**Chronic diseases/ Non-communicable diseases (NCDs)**	Non-communicable diseases (NCDs), also known as chronic diseases, are not passed from person to person. They are of long duration and generally slow progression. The four main types of noncommunicable diseases are cardiovascular diseases (like heart attacks and stroke), cancers, chronic respiratory diseases (such as chronic obstructive pulmonary disease and asthma) and diabetes (http://www.who.int/mediacentre/factsheets/fs355/en/).
**Nurses**	Any qualified nurse working as a substitute to a primary care physician focused on Self- management support for chronic disease. This could include: nurse practitioners, clinical nurse specialists, advanced practice nurses, practice nurses, registered nurse, etc. As the job title, education, and experience of nurses varies considerably among and within countries. We did not select nurses by virtue of their job title but, based on the description of interventions and competencies (experience/training/qualifications) we categorized nurses’ roles into: (a) advanced practice nurse (APN) for example nurse specialist, nurse case manager and nurse practitioner and (b) registered nurse. We focused our interest mainly stressing the difference between basic and advanced level of nurse qualifications, to promote future comparison of job profile and a more efficient nurses insertion in the healthcare workforce.
**Community setting**	Primary care settings included patient home and community-based facilities. These were nurse clinics, general medicine clinics, primary care practices, family medicine centers, primary care clinics, community and municipal hospitals. In-hospital based care and discharge planning program from hospital were excluded.

Studies aimed to evaluate the efficacy of a nurse-led self-management support intervention, compared to the usual care, to improve observer-reported outcomes (OROs) [29, 30]–particularly clinical outcomes–and patient-reported outcomes (PROs) [30, 31]as primary outcomes. We excluded studies that evaluated interventions in which nurses were only involved in medical assessment or therapy optimization and studies that enrolled patients with mental disorders. To ensure maximum retrieval, two reviewers with different skills in bibliographic search methodology and in nursing chronic disease management, searched together for RCTs in MEDLINE (to July 2016) using the strategy reported in S1 File. Additional searches in CINAHL, Scopus and Web of Science were carried out using similar syntax; experts were consulted and bibliographies of relevant articles were reviewed. Bibliographic search was restricted to studies reported in English. Each citation found in the databases was reviewed independently by two authors via a titles-first approach to obtain records for the abstract screening.

### Study selection and quality assessment

Two reviewers independently reviewed the abstracts obtained in the search and retrieved the full text article of those that met the inclusion criteria. In cases of disagreement, full text article for review was retrieved. The methodological quality of the RCTs was assessed independently by two reviewers using the risk of bias approach described in the Cochrane Handbook [32]. Random sequence generation, allocation concealment, blinding, incomplete outcome data, selective outcome reporting and other potential sources of bias were described and assessed. Any disagreements about methodological quality were resolved by discussion and, if necessary, a third reviewer was involved.

### Data extraction

Two reviewers performed data extraction and data entry independently, in duplicate. Differences in data extraction were discussed and if necessary resolved by a third reviewer. A standardized form was used to abstract the following data: bibliographic details; population demographics; interventions; patient condition (diabetes, cardiovascular diseases (CVD), multichronic conditions); type of nurses employed in the study (RN: registered nurse; APN: advanced practice nurse); availability of specific training for the nurses that provide the intervention; type of intervention (face-to-face; telephone/telemedicine; mixed); duration of the intervention; study sample size; outcome data (continuous or binary).

### Data synthesis

A rating system, based on the methodological quality of the studies and on the consistency of the findings [33, 34], was used to assess the strength of the evidence for OROs and PROs. The results were synthesized and assigned one of the following three levels of scientific evidence:

- strong evidence: provided by generally consistent findings, supporting the hypotheses, in multiple high-quality studies;
- moderate evidence: provided by generally consistent findings, supporting the hypotheses, in one high quality study and one or more moderate quality studies, or in multiple moderate quality studies;
- insufficient evidence: only one study available or inconsistent findings in multiple studies.

To summarize continuous data, the pooled mean difference (MD) and 95% confidence interval (CI) were calculated [35]. A random effect approach was chosen for all analyses to account for between-study variance [36]. The fixed-effects model [37] was also used to check the level of agreement with random effects conclusions. The I^2^ metric, which describes the percentage of total variation across studies that was due to heterogeneity rather than sample error (chance) [38], was used to test for heterogeneity. If I^2^ was ≥60%, a sensitivity analysis was performed by removing the studies contributing to the heterogeneity. Results of studies reported in multiple articles were included once in each meta-analysis. Presence of publication bias was assessed through funnel plot graph.

Given the highly diverse nature of the studies analysed, several stratified meta-analyses were carried out to explore the efficacy in subgroups; meta-analyses were also carried out in the absence of statistical heterogeneity. In particular, we analyzed the effect of the following stratification factors: patient condition (diabetes, cardiovascular diseases (CVD), multichronic conditions); type of nurses employed in the study (RN: registered nurse; APN: advanced practice nurse); availability of specific training for the nurses that provide the intervention; type of intervention (face-to-face; telephone/telemedicine; mixed); duration of the intervention (≤6 months; >6 months); study sample size (≤200; >200); attrition rate (<20%; ≥20%); allocation concealment (clearly stated; undefined/absent).

All meta-analyses were performed using RevMan software, version 5.2 (Cochrane Collaboration, Oxford, UK, 2012). Reporting was made following the PRISMA Statement guidelines (see S2 File for the Checklist).

## Results

### Main characteristics of the included studies

**Fig 1:**
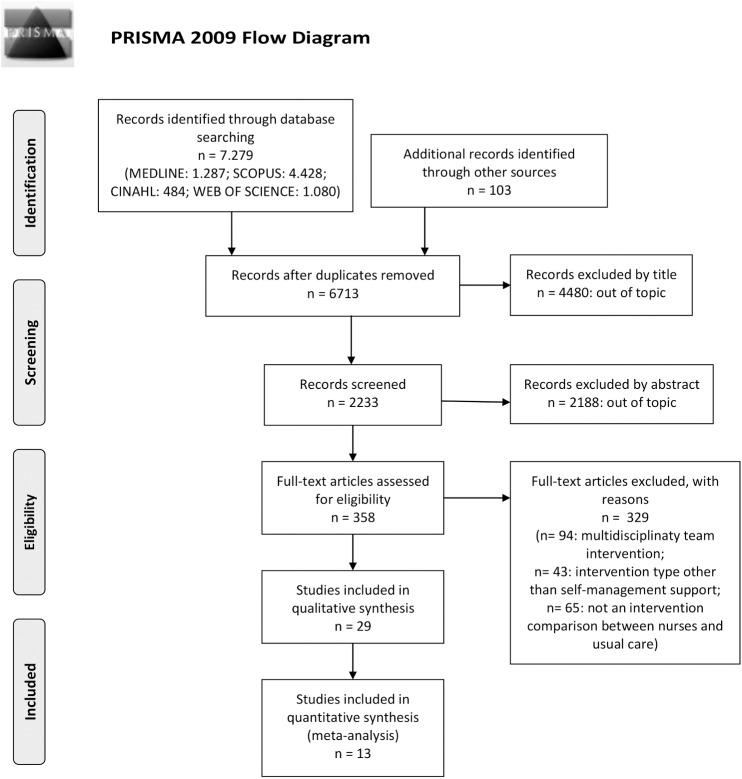
Flow diagram of the study selection process. *From*: Moher D, Liberati A, Tetzlaff J, Altman DG, The PRISMA Group (2009). Preferred Reporting Items for Systematic Reviews and Meta-Analyses: The PRISMA Statement. PLoS Med 6(7): e1000097. doi: 10.1371/journal.pmed1000097.

Of the 7,279 papers initially retrieved (Fig 1) 29, that describe the results of 23 studies, met our inclusion criteria (see S1 Table for a summary of the main characteristics and an overall quality score of the studies included in the review). A summary of the type of intervention and primary outcomes measured in each study is reported in Table 2.

**Table 2:** Summary characteristics of the intervention of included studies.

*Author*, *Year*, *Country*	*Intervention/Setting*	*Disease*	*Primary Outcomes*
**Bischoff et al., 2012, The Netherlands**	The self-management program consisted of paper modules and a written exacerbation action plan. The practice nurse applied the program to the individual patient in two to four sessions of approximately one hour each, scheduled in four to six consecutive weeks, followed by telephone calls/General practice.	COPD	Quality of Life
**Bosworth et al., 2005, USA**	Telephone contacts every 2 months for 24 months. The nurse delivers both tailored and standard information in nine modules/Primary care clinics.	Hypertension	Primary outcome not evaluated (Only secondary outcome reported)
**Bosworth et al., 2009, USA**	See Bosworth 2005	Hypertension	BP control
**Boyd et al., 2010, USA**	A comprehensive assessment at home, creation and maintenance of an evidence based "Care Guide" (care plan) and an Action Plane (patient's self-care plane), monthly monitoring, coaching for self-management, smoothing transition into and out of hospitals, coordinating all providers of care, educating and supporting family caregivers and accessing community resources/Patient’s home	Multichronic	Patient Assessment of Chronic Illness Care (Goal setting; Coordination of care; Decision support; Problem solving; Patient activation; Aggregate quality)
**Cooper et al., 2008, UK**	Health educational program–LAY (Look After Yourself) for physical activities and exercise, relaxation, health topics. 2 hours sessions weekly for 8 weeks/Hospital diabetes outpatient clinics and General Practice center	Diabetes	HbA1c
**Delaney et al., 2008, UK**	Attendance of secondary prevention nurse-led clinics during which patients’ symptoms and treatment were reviewed, use of aspirin promoted, blood pressure and lipid management reviewed, lifestyle factors assessed and, if appropriate, behavioral changes negotiated/Secondary prevention nurse-led clinics in general practice	Coronary heart disease	Total Mortality; Coronary events
**Denver et al., 2003, USA**	The hypertension nurse emphasized the need for tight BP control, gave non-pharmacological advice for healthy living, and (if necessary) discussed problems regarding side effects of existing antihypertensive treatment/Outpatient nurse-led clinics from the hospital diabetes clinic	Multichronic	SBP, DBP
**Gabbay et al., 2013, USA**	The intervention group met individually within their primary care clinic with their nurse case managers at baseline, 2 weeks, 6 weeks, 3 months, 6 months, 12 months, and at least every 6 months thereafter. Visits were approximately 1-hour long. Participants intervention group could also contact their NCM (nurse case manager) by phone calls or e-mails between visits when appropriate/Primary care clinic	Diabetes	HbA1c; LDL; SBP; DBP; Diabetes-related emotional distress; Satisfaction with the diabetes regimen; Impact of diabetes on quality of life; Depression symptoms; Self-care activities
**Galbreath et al., 2004, USA**	Telephone education. In the event that a patient was thought to be unstable by the disease manager, face-to-face evaluation with a home healthcare nurse could be arranged. Initial call frequency was weekly, with a transition to monthly/Patient’s home	Chronic heart failure	Total mortality
**Garcia-Peña et al., 2001, Mexico**	Regular home visits from a nurse over 6 months with blood pressure measurement, information from the baseline health check, discussion about possible healthier lifestyle changes, suggestion of different alternative ways to achieve the changes with negotiation of specific target. Review of the pharmacological treatment and adherence encouragement/Patient’s home	Hypertension	Reduction in SBP; Reduction in DPB
**Gary et al., 2003, USA**	Home telephonic calls. The model incorporates critical constructs from adult learning, social support, and behavior modification theories and health services research such as predisposing, reinforcing, and enabling factors/Patient’s home	Diabetes	HbA1c
**Goudswaard et al., 2004, The Netherlands**	One-to-one sessions focused on: general information on diabetes (monitoring home blood pressure and home glucose levels); reinforcing compliance with actual medication; importance of physical exercise and losing body weight; and nutritional advice. During the 6-month period, six sessions were given, at intervals of 3–6 weeks/General practice	Diabetes	HbA1c
**Ishani et al., 2011, USA**	Patients, in collaboration with the study nurses, established lifestyle modification goals and developed personal action plans. Contacts every 2 weeks initially and for the frequency of contact to decrease as the patient achieved home BP and glucose goals. The study duration was 12 months/Patient’s home	Diabetes	% achieving BP 130/80mmHg; LDL 100mg/dL; HbA1c, 8.0%
**Krein et al., 2004, USA**	Patient contact occurred primarily by telephone, although face-to-face visits could be arranged. Case managers were directed to encourage patient self-management, including diet and exercise; provide reminders for recommended screenings/tests; help with appointment scheduling; monitor home glucose and home blood pressure levels; and identify and initiate medication and dose changes as needed/Outpatient case management	Diabetes	HbA1c; LDL; SBP; DBP
**Lee et al., 2007, UK**	Six-month community-based walking intervention delivered by the public health nurse. A series of regular individual contacts was provided through telephone and face-to-face visits/Local community activity centers and patient’s home	Hypertension	Change in SBP; Reduction in DBP
**Murchie et al., 2003, UK**	See Delaney et al., 2008	Coronary heart disease	Use of secondary prevention (aspirin, BP managemet, lipid management, healthy diet, exercise, non-smoking); Total Mortality; Coronary events
**Murchie et al., 2004, UK**	See Delaney et al., 2008	Coronary heart disease	Quality of Life; Anxiety; Depression; Chest pain; Worsening chest pain
**Piette et al., 2000, USA**	Automated telephone calls were used to deliver targeted and tailored self-care education messages/General medicine clinic	Diabetes	Glucose self-monitoring; Foot inspection self-monitoring; Weight self-monitoring; Perceived glycemic control; Diabetes-related symptoms; HbA1c; Serum Glucose
**Rudd et al., 2004, USA**	Nurse counseling at baseline on correct use of the automated home BP device, regular return of the automatically printed BP reports, tips for enhancing drug adherence, and recognition of potential drug side effects. Follow up phone contacts at 1 week and at 1, 2, and 4 months/Patient’s home	Hypertension	Reduction in DBP; Reduction in SBP; Medication adherence; Antihypertensive medications changes
**Shea et al., 2006, USA**	Home telemedicine unit (HTU). Nurse case managers were trained in diabetes management and in the use of computer-based case management tools to facilitate interactions through videoconferencing with patients/Patient’s home	Diabetes	HbA1c; SBP; DBP; LDL; Total Cholesterol
**Shea et al., 2009, USA**	See Shea et al., 2006	Diabetes	HbA1c; SBP; DBP; LDL
**Sisk et al., 2006, USA**	Face-to-face visit at baseline, home telephone follow-up/Community hospitals	Chronic heart failure	Hospitalizations; Functioning (physical component); Mortality
**Taylor et al., 2003, USA**	All intervention patients were asked to attend a 1- to 2-h group class that met once a week for 4 weeks. Telephone follow-up calls/Primary care center and patient’s home	Multichronic	HbA1c; Total cholesterol; LDL; HDL; Triglycerides; Glucose; SBP; DBP; BMI; dilated eye exam; Flu shot; Foot exam; Dental exam; Quality of life; Depression; Patients satisfaction; Physician satisfaction; physician’s visits; Hospitalization; Emergency room
**ter Bogt et al., 2009, The Netherlands**	Four individual visits and one feedback session by telephone in the first year/Patient’s home	Multichronic	Outcomes evaluated in subgroups of women and men: Weight; Weight %; Waist; SBP; DBP; Total cholesterol; HDL; LDL; Fasting glucose; Weight losers and stabilizers
**ter Bogt et al., 2011,The Netherlands**	See ter Bogt et al., 2009	Multichronic	Weight; Weight %; BMI; Waist; SBP; DBP; Total cholesterol; HDL; LDL; Fasting glucose; Impaired fasting glucose; Weight losers and stabilizers; Weight regainers
**Tonstad et al., 2007, Norway**	Monthly meetings with the nurse for 6 months. The initial session lasted for 60 min and subsequent sessions lasted for 30 min/Patient’s home	Hypertension	Reduction in DBP; Reduction in SBP; Number of Metabolic syndrome risk factors (glucose, Hb1Ac, triglyceride concentrantions, total cholesterol, waist circumference, weight)
**Walters et al., 2013, Australia**	Telephone calls 16×30 min over 12 months, with increasing time between calls/Patient’s home	COPD	Quality of life
**Woollard et al., 2003, Australia**	The high-level intervention group were counselled in individual sessions up to 60 min every month over a period of 12 months. Participants were provided with a personalized educational manual developed to support the cognitive behavioral approach/General practice	Multichronic	Total Energy intake; Total Fat; Satured Fat; Polyunsatured Fat; Monousatured Fat; Sodium; Potassium; Fibre; Alcohol; Total cholesterol; LDL; HDL; triglycerides; n3/n6 fatty acids; BMI; Weight; Waist to hip ratio
**Woollard et al., 2003b, Australia**	See Wollard et al., 2003a	Multichronic	SBP; DBP; 24h SBP; 24h DBP; Awake SBP; Awake DBP; Asleep SBP; Asleep DBP; 24h Heart rate; BMI; Weight; Energy intake; Fibre Intake; Alcohol Intake; Physical activity; Fasting blood sugar; glycated hemoglobin; Urinary sodium; Urinary Potassium

The studies were published from 2000 to 2013, mainly in the USA (15), the UK (5) and the Netherlands (4). Overall, 10,162 patients were enrolled in the 23 studies (range: 51–1665), seven of which enrolled fewer than 200 patients. Six papers [39–44] reported analyses of previous studies [45–49], which extended the follow-up and/or took into account different outcomes; these were included in the meta-analyses as appropriate. Patients’ mean age was reported in all studies, ranging from 55.5 [25] to 77.2 [26] for the experimental group and from 54.8 [25] to 78.1 [26] for the control group. The majority of the papers assessed the efficacy of the interventions among patients affected by cardiovascular diseases (11), diabetes (9) or multichronic conditions (7). Only two papers took into account patients with COPD. Interventions were mainly carried out at patients’ homes (10 studies) and in general practices (five studies) by APNs (13 studies) and RNs (10 studies); the nurses were specially trained in 15 studies. It is interesting to note that self-management skills were appropriately assessed in patients by validated tools in only five studies.

The methodological quality was high in nine studies and moderate in another nine (S2 Table). Only one paper fulfilled all the criteria for reducing risk of bias. Eight studies failed to report only one of the criteria. Nine papers out of 29 did not report on the methods used to randomly allocate patients to groups and in 20 and 11 cases the allocation concealment and the blinding, respectively, were not sufficiently detailed or were clearly absent. Five studies were at high risk of bias for attrition.

### Observer-reported outcomes

#### Blood pressure levels

Overall, 12 studies [24, 25, 39, 43, 44, 48, 50, 51–55] evaluated the levels of systolic blood pressure (SBP) as a primary outcome–on a total of 5,671 patients–showing strong evidence. Seven studies [24, 43, 48, 50, 52, 53, 55] out of 12 found that SBP levels were significantly lower in the experimental groups than in the control groups (Table 3); in particular, all studies with shorter interventions [24, 50, 52, 53] showed significant results.

**Table 3:** Findings of the impact of nurse led-self management interventions on Observer Related Outcomes (OROs) and Patient Related Outcomes (PROs).

Category	Reference	Result	Evidence
*Observer Reported Outcomes*	* *	* *	* *
Systolic blood pressure	Denver 2003	+	Strong
	Gabbay 2013	+	
	Garcia-Peña 2001	+	
	Krein 2004	n.s.	
	**Lee 2007**	+	
	**Rudd 2004**	+	
	**Shea 2006**	+	
	Shea 2009	+	
	Taylor 2003	n.s.	
	ter Bogt 2011	n.s.	
	Tondstad 2006	n.s.	
	Wollard 2003	n.s.	
Diastolic blood pressure	Denver 2003	n.s.	Strong
	Gabbay 2013	n.s.	
	Garcia-Peña 2001	+	
	Krein 2004	n.s.	
	**Lee 2007**	n.s.	
	**Rudd 2004**	+	
	**Shea 2006**	+	
	Shea 2009	+	
	Taylor 2003	n.s.	
	ter Bogt 2011	n.s.	
	Tondstad 2006	n.s.	
	Wollard 2003	n.s.	
HbA1c	Cooper 2008	n.s.	Strong
	Gabbay 2013	n.s.	
	Gary 2003	n.s.	
	**Goudswaard 2003**	+	
	Krein 2004	n.s.	
	Piette 2000	n.s.	
	**Shea 2006**	+	
	Shea 2009	+	
	Taylor 2003	+	
	Tondstad 2006	n.s.	
	Wollard 2003	n.s.	
Total cholesterol	Taylor 2003	+	Insufficient
	ter Bogt 2011	n.s.	
	Tondstad 2006	n.s.	
	Wollard 2003	n.s.	
LDL cholesterol	Gabbay 2013	n.s.	Moderate
	Krein 2004	n.s.	
	**Shea 2006**	+	
	Shea 2009	n.s.	
	Taylor 2003	+	
	ter Bogt 2011	n.s.	
	Wollard 2003	n.s.	
Fasting serum glucose	Piette 2000	+	Insufficient
	Taylor 2003	n.s.	
	ter Bogt 2011	+	
	Tondstad 2006	n.s.	
	Wollard 2003	n.s.	
Triglycerides	Taylor 2003	n.s.	Insufficient
	Tondstad 2006	+	
Total Mortality	Delaney 2008	n.s.	Insufficient
	Galbreath 2004	+	
	Murchie 2003	+	
	**Sisk 2006**	n.s.	
*Patient Reported Outcomes*	* *	* *	* *
Quality of life	**Bischoff 2012**	n.s.	Insufficient
	Gabbay 2013	n.s.	
	Murchie 2004	+	
	Walters 2013	n.s.	

+: Statistically significant results in favor of the intervention

n.s.: not statistically significant results. The high quality studies are in bold. Levels of evidence: Strong, Moderate, Insufficient.

The majority of effective interventions were carried out by advanced nurses/case managers [43, 48, 52, 53, 55]. A variety of intervention techniques were used: four out of the seven effective studies used face-to-face studies [24, 50, 55] or face-to-face/telephone [53] nurse visits; these were delivered at the patient’s home [50, 53], in nurse-led clinics [24], at local community activity centres [53] or in primary care clinics [55].

**Fig 2:**
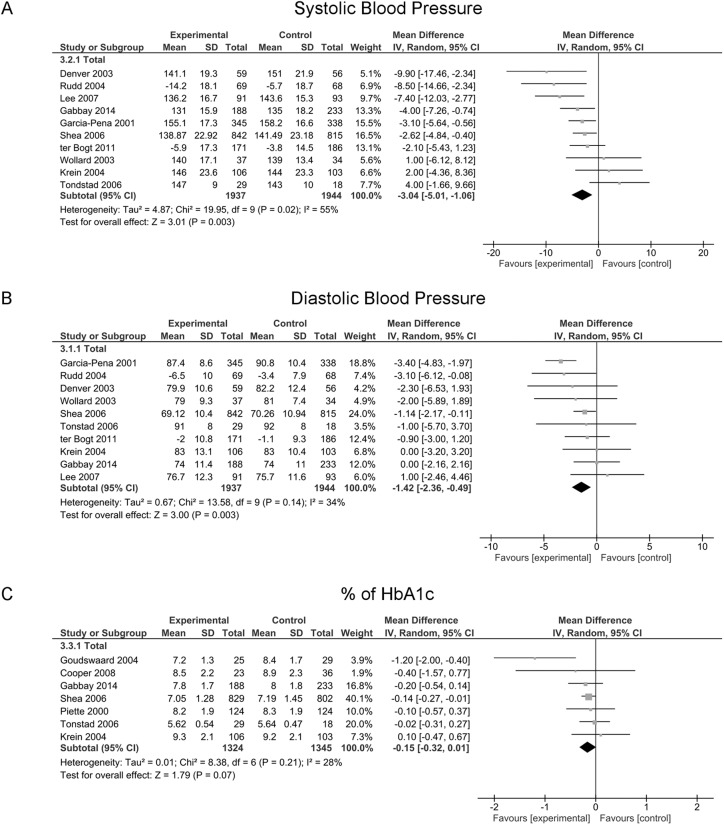
Comparison of the effect of nurse-led support interventions and usual care on the reduction of some Observer Related Outcomes (OROs): Systolic Blood Pressure, Diastolic Blood Pressure and Hb1Ac*. *Only for diabetic patients.

A meta-analysis on SBP reduction was carried out on 10 studies [24, 39, 44, 48, 50–55], involving a total of 3,881 patients. The pooled MD was -3.04 (95% CI -5.01 to -1.06) in favour of the interventions, with significant heterogeneity between studies (I^2^ = 55%, p = 0.02) (Fig 2).

Meta-analyses of subgroups showed a statistically significant effect if the interventions were delivered to diabetic patients (MD -2.56, 95% CI -4.82 to -0.31), if an APN was employed (MD -3.57, 95% CI -6.36 to -0.78), if the nurses were specially trained (MD -2.81, 95% CI -4.30 to -1.32), if the studies had a sample size greater than 200 patients (MD -0.13, 95% CI -0.25 to -0.01) and if the allocation concealment was not clearly defined (MD -2.54, 95% CI -5.04 to -0.56). Stratification by type of intervention failed to show a significant effect of any specific intervention. Neither length of intervention nor attrition rate influenced the results, which remained significant in favour of intervention (Table 4).

**Table 4:** Meta-analysis of the reduction of blood pressure levels stratified by level and training of employed nurses; type and duration of the intervention; study size; attrition rate; allocation concealment.

Blood Pressure									
*Subgroup*	*RCTs*, *N*	*Intervention group*, *N*	*Control group*, *N*	*SBP*			*DBP*		
				*MD (95% IC)*	*p*	*I*^*2*^, *%*	*MD (95% IC)*	*p*	*I*^*2*^, *%*
**Diseases**									
CVD	4	534	517	-3.74 (-8.28, 0.81)	0.11	75	**-2.09 (-4.11, -0.07)**	**0.04**	49
Diabetes	3	1136	1151	**-2.56 (-4.82, -0.31)**	**0.03**	26	-0.86 (-1.75, 0.03)	0.06	0
Multichronic	3	267	276	-3.26 (-8.40, 1.89)	0.21	57	-1.33 (-3.03, 0.36)	0.12	0
**Employed Nurses**									
RN	5	658	679	-2.39 (-5.74, 0.96)	0.16	62	**-1.90 (-3.57, -0.23)**	**0.03**	42
APN	5	1279	1265	**-3.57 (-6.36, -0.78)**	**0.01**	57	**-1.05 (-1.88, -0.23)**	**0.01**	0
**Specific training**									
Trained	7	1758	1777	**-2.81 (-4.30, -1.32)**	**<0.001**	18	**-1.56 (-2.63, -0.48)**	**0.004**	47
Untrained	3	179	167	-4.28 (-12.58, 4.01)	0.31	83	-0.49 (-2.81, 1.84)	0.68	0
**Type of intervention**									
Face-to-face	5	641	632	-1.89 (-5.13, 1.36)	0.25	59	**-2.41 (-3.54, -1.28)**	**<0.001**	5
Telephone/Telemedicine	2	911	883	-4.83 (-10.41, 0.75)	0.09	68	-1.59 (-3.20, 0.02)	0.05	31
Mixed	3	385	429	-3.62 (-8.04, 0.79)	0.11	64	0.21 (-1.38, 1.80)	0.79	0
**Duration**									
≤6 months	5	593	573	**-4.66 (-8.87, -0.45)**	**0.03**	73	**-2.25 (-3.88, -0.62)**	**0.007**	32
>6 months	5	1344	1371	**-2.38 (-3.90, -0.86)**	**0.002**	0	**-0.91 (-1.71, -0.11)**	**0.03**	0
**Study size**									
≤200	3	157	142	-4.60 (-13.68, 4.48)	0.32	83	**-2.44 (-4.61, -0.26)**	**0.03**	0
>200	7	1780	1802	**-2.91 (-4.43, -1.40)**	**<0.001**	23	**-1.19 (-2.33, -0.06)**	**0.04**	51
**Attrition rate**									
<20%	7	1541	1491	**-3.42 (-6.25, -0.58)**	**0.02**	67	**-1.68 (-2.93, -0.43)**	**0.008**	45
≥20%	3	396	453	**-2.68 (-4.89, -0.46)**	**0.02**	0	-0.66 (-2.06, 0.74)	0.36	0
**Allocation concealment**									
Clearly stated	2	933	908	-4.56 (-9.16, 0.04)	0.05	70	-0.73 (-2.38, 0.92)	0.38	26
Undefined/absent	8	1004	1036	**-2.54 (-5.04, -0.56)**	0.05	57	**-1.71 (-2.86, -0.56)**	**0.004**	31

The same 12 studies [24, 25, 39, 43, 44, 48, 50–55] explored the effect on diastolic blood pressure (DBP) levels in a total of 5,671 patients with strong evidence (Table 3). Ten studies with 3,881 patients in total were included in the meta-analysis on the reduction in DBP [24, 39, 44, 48, 50–55]. A statistically significant reduction in DBP was found for the whole group (MD -1.42, 95% CI -1.42 to -0.49) with no statistically significant heterogeneity between studies (I2 = 34%, p = 0.14) (Fig 2). The analysis of the funnel plot showed a lack of studies with large sample size and high effect measures.

An attempt was made to identify possible influencing factors using stratified meta-analyses. A statistically significant effect was shown for interventions on patients with CVD (MD -2.09, 95% CI -4.11 to -0.07), specific training of nurses (MD -1.56, 95% CI 2.63–0.48), face-to-face interventions (MD -2.41, 95% -3.54 to -1.28), attrition rate lower than 20% (MD -1.68, 95% CI -2.93 to -0.43) and unclear presence of allocation concealment (-1.71, 95% CI -2.86 to -0.56). Stratification by type of nurse employed, by sample size and by duration of intervention did not influence the results, which remained significant in all subgroups (Table 4).

#### HbA1c

Of the 29 included studies, 11 [25, 39, 43, 48, 51, 54–59] investigated HbA1c levels as a primary outcome in diabetic patients, resulting in strong evidence of the efficacy of intervention. Overall, these studies included 4,207 patients. The levels of HbA1c were significantly lower in the experimental groups than in the control groups in four studies [25, 43, 48, 58] (Table 3). The two studies with statistically significant results and high methodological quality were based on one-to-one sessions with patients led by a skilled diabetes RN [58] and on telemedicine and videoconferencing carried out by specially trained nurses [48].

The results of seven studies [48, 51, 54–56, 58, 59], involving 2,669 patients, were useful for pooling data. The MD showed a reduction in HbA1c of 0.15 in favour of the experimental group (95% CI -0.32 to 0.01) with a heterogeneity of I^2^ = 28, p = 0.21 (Fig 2). The funnel plot showed that the results were based mainly on small studies with low-effect measures.

After stratification, statistical significance was shown for specific training of nurses (MD -0.13, 95% CI -0.25 to -0.01), intervention by telephone/telemedicine (MD -0.14, 95% CI -0.27 to -0.01), intervention length >6 months (MD -0.13, 95% CI -0.25 to -0.01) and a sample size of >200 people (MD -0.13, 95% CI -0.25 to -0.01). Stratification by type of nurse employed, attrition rate and presence of allocation concealment failed to show significant differences between intervention and control (Table 5). Moderate or insufficient evidence was obtained for the reduction of total cholesterol, LDL cholesterol, triglycerides and fasting serum glucose (Table 3).

**Table 5:** Meta-analysis of the reduction of HbA1c levels in diabetic patients stratified by level and training of employed nurses; type and duration of the intervention; study size; attrition rate; allocation concealment.

Hb1Ac						
*Subgroup*	*RCTs*, *n*	*Intervention group*, *N*	*Control group*, *N*	*MD (95% IC)*	*p*	*I*^*2*^, *%*
**Employed Nurses**						
RN	4	366	404	-0.24 (-0.58, 0.09)	0.16	60
APN	3	911	894	-0.11 (-0.29, 0.07)	0.22	5
**Specific training**						
Trained	4	1099	1127	**-0.13 (-0.25, -0.01)**	**0.03**	0
Untrained	3	178	171	-0.32 [-0.86, 0.22]	0.24	73
**Type of intervention**						
Face-to-face	3	77	83	-0.49 (-1.29, 0.32)	0.23	73
Telephone/Telemedicine	2	953	926	**-0.14 (-0.27, -0.01)**	**0.04**	0
Mixed	2	247	289	-0.02 (-0.49, 0.45)	0.94	49
**Duration**						
≤6 months	3	77	83	-0.49 (-1.29, 0.32)	0.23	73
>6 months	4	1200	1215	**-0.13 (-0.25, -0.01)**	**0.03**	0
**Study size**						
≤200	3	77	83	-0.49 (-1.29, 0.32)	0.23	73
>200	4	1200	1215	**-0.13 (-0.25, -0.01)**	**0.03**	0
**Attrition rate**						
<20%	6	1089	1065	-0.14 (-0.38, 0.09)	0.24	47
≥20%	1	188	233	-0.20 (-0.54, 0.14)	0.24	n.a.
**Allocation concealment**						
Clearly stated	2	854	831	-0.59 (-1.62, 0.43)	0.26	85
Undefined/absent	6	529	570	-0.05 (-0.23, 0.13)	0.57	0

#### Total mortality

Three studies [45, 60, 61], with an overall sample size of 2,564 patients, evaluated total mortality. The study of Delaney et al. [41] used the same population and intervention as Murchie et al. [45] but considered the results from 10 years of follow-up. For all four studies the total number of deaths in the experimental groups was lower than in the control groups, reaching statistical significance in two studies [45, 60] (Table 3); these studies were based on interventions lasting 12 months [45] or longer [60] on patients with coronary heart disease or chronic heart failure led by RNs [45] or APNs [60, 61]. Educational interventions were based on face-to-face visits carried out at nurse-run clinics [45] or hospital [61] with telephone follow-up [60, 61].

#### Multiple clinical outcomes

Only one study [62] evaluated as a primary outcome the simultaneous reaching of a threshold in BP levels, LDL serum levels and percentage of HbA1c, taking into account 556 patients. A significantly higher percentage of patients in the intervention group reached the goals compared to the control group. The intervention consisted of an initial personal meeting with a nurse case manager, followed by follow-up telephone calls.

### Patient reported outcomes

#### Quality of life

Three studies [40, 63, 64] included changes in quality of life–evaluated with SF-36 [40, 64] or other questionnaires related to the specific disease aim of the study [63, 64]–as a primary outcome, but there was insufficient evidence of a significant effect. The overall scores of the questionnaires were analyzed. For two studies [40, 64] the overall scores in the experimental groups were higher rather than the control groups, but this result was only significant for the study of Murchie et al. [40] (Table 3). Educational interventions were based on face-to-face visits [40, 63] or telephone health mentoring [64] led by RNs [40, 64] or APNs [63].

## Discussion and conclusions

Primary care systems across the world are facing the challenge of an ageing population and an associated increase in the number of chronic patients [65, 66], leading to a growing demand for a kind of care [67] that meets emerging needs, reduces the costs of hospital-based ambulatory care and prevents avoidable hospital use by the provision of more appropriate care systems. In this context, the rational redistribution of tasks among health workforce teams–namely task shifting [15]–as a means of addressing this public health issue represents a potentially winning strategy. More particularly, serious attention has been payed to the support of patient self-management, since it can improve patient self-efficacy [8, 68], disease-related behaviors and, finally, enhance patients’ functional and health status [8, 69, 70]. Among health professionals, nurses can play a pivotal role in the delivery of self-management support interventions, particularly in areas of medical workforce shortage. This policy development clearly brings with it the need to continually seek updated evidence about the roles that nurses can undertake, their clinical effectiveness and cost-effectiveness in these roles, as well as patient satisfaction.

According to our systematic review and meta-analysis, nurse-led self-management support interventions in chronic care community programs have a positive impact on some OROs, such as a reduction in the levels of HbA1c, DBP/SBP and, to a lesser extent, LDL, particularly in patients with diabetes and CVD. Effects on other outcomes such as serum levels of total cholesterol, fasting serum glucose levels and triglycerides, as well as quality of life and all-causes mortality, remain inconclusive.

Diabetes and CVD are among the diseases that can most benefit from patient self-management. Empowering patients to manage their own diseases and fostering patient-centered activities can effectively reduce complications or reactivation of diseases that can shorten length of life and reduce autonomy. Self-management training in type 2 diabetes has evolved since the didactic primarily interventions of the 1970s into the empowerment models of the 1990s [69, 71]. Such a transformation has led to better glycemic control [69]. Our results confirm this and suggest also that trained nurses can effectively administer self-management support interventions to type 2 diabetes patients [25, 43, 48, 58]. A study published in 2004 showed that a nurse-led education intervention led to the improvement of glycemic control and a delay in the requirement for insulin therapy in patients treated with oral hypoglycemic therapy [58]. Moreover, our results show that nurse-led telemedicine interventions can also have a positive effect by reducing HbA1C levels [43, 48]. The remote monitoring and transmission of physiological data facilitate contact with a health care professional via telephone or video, while disease-specific education guarantees the reinforcement of self-management behaviors [72]. More difficulties were encountered in reducing serum levels of LDL [25, 39, 43, 48, 55] and triglycerides [25] in patients with diabetes. This is of particular interest since LDL oxidation does not decrease after improvement in metabolic control in type 2 diabetes [73]. Together with hypertriglyceridemia, LDL oxidation is involved in the pathogenesis of the so-called metabolic syndrome, which is associated with increased risk of CVD and for which lifestyle modification is an important therapeutic strategy [74]. Therefore, developers of educational interventions should focus on general knowledge of diabetes, adherence to medication, lifestyle changes and, if possible, self-monitoring of blood glucose [75].

With respect to CVD, the results of our meta-analysis also show that nurses can be more effective than the usual care-in-the-community systems in improving blood pressure control, eventually leading to reduced blood pressure levels. This positive effect is clearer when nurses are specially trained and is more significant among diabetes patients for SBP levels and among CVD patients for DBP levels. Face-to-face interventions seem to be more effective, at least for the reduction of DBP levels, even though nurses also significantly improve self-management behavior by telephone interventions [47].

Nurse-led intervention is less effective at improving clinical outcomes in multi-chronic patients [24–26, 39, 44, 46, 49] probably because of the subjective and objective barriers to good self-management associated with this condition. Indeed, comorbidity has been mentioned in previous studies as a limit to self-care [76, 77]. A semi-structured interview study concluded that major barriers to self-care for people with more than one chronic disease are mainly linked to the combined effects of multiple conditions or to a single dominant disease making the management of the other conditions difficult. Other barriers were identified as a lack of patient knowledge about their conditions, financial constraints, low self-efficacy, inadequate communication with providers, the need for or use of social support and finally various logistical issues [78]. Another qualitative study, which used patient focus groups, placed much more emphasis on the role of physician communication and family support as barriers to the self-management of their chronic conditions [79]. Clearly, self-care interventions for people with multiple chronic diseases must be tailored to patients’ real needs, since they are likely to be more effective if targeted at particular risk factors or specific functional difficulties [80].

The finding that the benefits of nurse-led intervention to support patient self-management disappear when nurses are not specially trained is one of the most important results of this meta-analysis. Ad hoc training seems to be more important than the role and general experience of the nurse. In fact, the results of the meta-analyses show that APNs are more effective than RNs only in reducing SBP levels. Provider training is recognized to be a key factor in the entire self-management support intervention process. Studies that evaluated the effectiveness of in-person training have reported generally positive results [81–83]. However, promising results also derive from web-based self-management training for health professionals: webinar-based training sessions can benefit participants’ self-beliefs and confidence [84].

Several studies have determined that, among health professionals, nurses are best placed to promote health and to deliver preventive programs within the primary care context [85, 86]. Their employment as providers of self-management support programs in primary care can further improve the health status of chronic patients, even if the task shifting from physicians to nurses in this particular area requires specific education and training. Further research on the efficacy of nurse-led self-management support programs must focus on long-term outcomes. Evidence on the effect of these programs on mortality and hospitalization rates is still insufficient or lacking. Moreover, the evaluation of patient self-efficacy in experimental studies that use reliable and valid instruments is urgently required.

Finally, the methodological quality of RCTs must be improved. In many cases, in the particular context of trials that evaluate the efficacy of nurse-led interventions vs. physician-led interventions, blind participation in the intervention is not always possible. This was often acknowledged in the included studies, but it was not always counterbalanced by appropriate allocation concealment that, in such cases, is universally recognized to reduce bias [87].

Our systematic review and meta-analysis have several weaknesses that must be taken into account. First of all, we included only publications in English and we did not search for grey literature. However, we made the literature search as widespread and inclusive as possible; primarily, we used electronic databases, but also screened the bibliographies of the retrieved articles for relevant publications. Second, one may argue that some clinical and physiological characteristics of the patients other than the educational interventions could influence the outcomes. To reduce this possibility to a minimum, we included only RCTs because of their lower risk of bias and we used restrictive inclusion and exclusion criteria to minimize heterogeneity among patient populations in terms of severity of disease, learning abilities and capacity to realize autonomously the activities of daily living. However, future research that includes non-randomised trials and/or observational studies are strongly recommended. Finally, we included different types of intervention. We decided to use this strategy because even though self-management support interventions differ in terms of target population, mode, format and content, it is clear that this variability in approach does not markedly affect outcomes [88]. Moreover, we made stratified analyses to account for some characteristics of the interventions that might affect the results.

In conclusion, self-management is a key focus of health policies for chronic disease control in many countries. Nurse-led self-management support interventions can be included in routine primary care activities, since specially trained nurses appear to be more effective than physicians in educating patients with diabetes and CVD in self-management of blood pressure and Hb1Ac in community settings. Future research should evaluate the efficiency of task shifting from physicians to nurses in community settings. Furthermore, trials with higher methodological quality and larger patient populations are urgently needed to assess the efficacy of self-management programs, since current evidence is based on very few large studies of mixed methodological quality.

## Supporting information

S1 File Research strategy and study eligibility criteria. (DOCX)

S2 File PRISMA 2009 Checklist. (DOC)

S1 Table Summary characteristics of participants and interventions of included studies. (DOCX)

S2 Table Methodological quality assessment. (DOCX)
