# Supplementary material for: Are community-based nurse-led self-management support interventions effective in chronic patients? Results of a systematic review and meta-analysis
Source: PLoS One. 2017 Mar 10;12(3):e0173617. doi: 10.1371/journal.pone.0173617 (PMC5345844; doi:10.1371/journal.pone.0173617)
Supplement: S1 File — (DOCX) [file pone.0173617.s001.docx]

**Research Strategy**

**#1 Medline, CINAHL, Web of Science**

((”Primary Health Care” OR “Chronic Disease” OR “Comorbidity” OR “Multimorbidity” OR ”Community Health Services” OR ”Complex Patient” OR “Ambulatory Care”) AND (“Nurse-Led Secondary Prevention” OR “Nurse Health Educator” OR “Nurse's Practice Patterns” OR “Promotion of Healthy Behaviors” OR “Modify Lifestyle” OR “Preventive Care” OR “Health Teaching” OR “Patient Education” OR “Caregiver Education” OR “Symptoms and Risk Factors Assessment” OR “Community Health Nurse” OR “Health Education” OR “Self Care” OR “Self Management Support” OR “Health Behavior” OR “Counseling” OR “Secondary Prevention” OR “Disease Management” OR “Health Promotion” OR “Teach-Back Communication” OR “Patient Care Management” OR “Primary Care Nursing” OR “Advanced Practice Nursing” OR “Case Management” OR “Nurse-Led Self Management Programme” OR “Nurse-Led Education” OR “Nurse-Led Follow Up Care” OR “Nurse-Led Telephone Follow Up” OR “Nurse Practitioners” OR “Nurse Clinicians” OR “Practice Nurse” OR “ Educational Therapy” OR “Patient Empowerment” OR “Health Coaching” OR “Motivational Interviewing” OR “Integrated Disease Management”) AND (“Physician-Led Secondary Prevention” OR “Physician Health Educator” OR “Physicians” OR “General Practitioners” OR “Primary Care Physicians” OR “Doctors”) AND (“Health Status Indicators” OR “Health Status” OR “Patient Compliance” OR “Life Style Changing” OR “Risk Reduction Behavior” OR “Self Efficacy” OR “Patient Satisfaction” OR “Patient Preference” OR “Quality of Life” OR “Life Style Changing” OR “Patients’ intentions for seeking care in the future” OR “Patient Medication Knowledge” OR “Mortality” OR “Hospital Admissions” OR “Patient Readmission**”** OR **“**Hospitalization”)

**#2 Scopus**

((”Primary Health Care” OR “Chronic Disease” OR “Comorbidity” OR “Multimorbidity” OR ”Community Health Services” OR ”Complex Patient” OR “Ambulatory Care”) AND (“Nurse-Led Secondary Prevention” OR “Nurse Health Educator” OR “Nurse's Practice Patterns” OR “Community Health Nurse” OR “Patient Care Management” OR “Primary Care Nursing” OR “Advanced Practice Nursing” OR “Case Management” OR “Nurse-Led Self Management Programme” OR “Nurse-Led Education” OR “Nurse-Led Follow Up Care” OR “Nurse-Led Telephone Follow Up” OR “Nurse Practitioners” OR “Nurse Clinicians” OR “Practice Nurse”) AND (“Promotion of Healthy Behaviors” OR “Modify Lifestyle” OR “Preventive Care” OR “Health Teaching” OR “Patient Education” OR “Caregiver Education” OR “Symptoms and Risk Factors Assessment” OR “Health Education” OR “Self Care” OR “Self Management Support” OR “Health Behavior” OR “Counseling” OR “Secondary Prevention” OR “Disease Management” OR “Health Promotion” OR “Teach-Back Communication” OR “ Educational Therapy” OR “Patient Empowerment” OR “Health Coaching” OR “Motivational Interviewing” OR “Integrated Disease Management”) AND (“Physician-Led Secondary Prevention” OR “Physician Health Educator” OR “Physicians” OR “General Practitioners” OR “Primary Care Physicians” OR “Doctors”) AND (“Health Status Indicators” OR “Health Status” OR “Patient Compliance” OR “Life Style Changing” OR “Risk Reduction Behavior” OR “Self Efficacy” OR “Patient Satisfaction” OR “Patient Preference” OR “Quality of Life” OR “Life Style Changing” OR “Patients’ intentions for seeking care in the future” OR “Patient Medication Knowledge” OR “Mortality” OR “Hospital Admissions” OR “Patient Readmission” OR “Hospitalization”))

**Study eligibility criteria**

***Inclusion criteria***

1. Randomized Controlled Trial
2. Interventions carried out in community settings
3. Patients >18 years old with a diagnosis of chronic disease (e.g. heart failure, diabetes, COPD, asthma) or multiple morbidity, in community setting, who had at least a hospitalization
4. Patients >18 years old with a diagnosis of chronic disease (e.g. heart failure, diabetes, COPD, asthma) or multiple morbidity cared in community setting, who had at least once an acute event
5. Secondary or tertiary prevention intervention
6. Experimental group intervention led by nurse Care Manager
7. Experimental group intervention led by nurse Case Manager
8. Experimental group intervention led by Registered Nurse/Practice Nurse/Nurse Specialist
9. Experimental group intervention led by Nurse Practitioner
10. Control group intervention led by Primary Care Physician/General Practitioner/Primary Community Physician
11. Telemedicine intervention led by registered nurse and General Practitioner
12. Self-sufficient patient able to learn and reproduce self-management support intervention
13. Patient enrolled during hospitalization for whom the largest part of the intervention is carried out in an out-patient/community setting

***Exclusion criteria***

1. Intervention provided in pediatric/gynecological/neonatal area
2. Intervention provided for illness exacerbation, acute illness or in emergency setting
3. Primary care intervention
4. Patients afflicted only by mental disorders, drugs addiction, alcoholism
5. Patients afflicted only by infectious diseases
6. Patients afflicted by neoplastic disease, cancer survivors or patient receiving palliative care
7. Studies in which the educational role is held by health professionals other than nurses or physicians (e.g. pharmacists, physiotherapists, community health workers)
8. Studies in which nurse/physician’s educational role cannot be distinguished from that of other health professionals (multidisciplinary team or nurse-physician collaboration) or is replaced by another type of intervention (e.g. electronic medical records, web-based tools, distribution of printed information material)
9. Studies in which the intervention is compared only between physicians
10. Studies in which the intervention is compared only between nurses
11. Studies related to organizational aspects of the primary care network
12. Qualitative studies
13. Studies related to health professional education
14. Clinical Guidelines or Clinical Descriptive studies
15. Studies without intervention comparison (non-experimental studies, descriptive studies, observational studies etc..)
16. No relevant studies on the research question
17. Commentaries, dissertation thesis
18. Studies not carried out in community settings (e.g. hospitalization)
19. Type of intervention different from self-management support
20. Studies in which the intervention in the control group is not conducted by the primary care physician (General Practitioner, Primary Community Physician)
21. Study population consisting of healthy individuals or subjects not afflicted by chronic diseases or afflicted by minor diseases (e.g. respiratory infections, lower back pain, diarrhea, urinary incontinence) or not able to realize activities of daily living (ADLs)
22. Education intervention carried out in a discharge planning from the hospital
