# Supplementary material for: Are community-based nurse-led self-management support interventions effective in chronic patients? Results of a systematic review and meta-analysis
Source: PLoS One. 2017 Mar 10;12(3):e0173617. doi: 10.1371/journal.pone.0173617 (PMC5345844; doi:10.1371/journal.pone.0173617)
Supplement: S1 Table — (DOCX) [file pone.0173617.s003.docx]

| *Author, Year Country* | *Nursing qualification* | *Specific Training* | *Self Managment Support Assessment* | *Intervention/Setting* | *Disease* | *Sample size* | *Results* | *Quality Score* |
| --- | --- | --- | --- | --- | --- | --- | --- | --- |
|  |  | *Y/N* | *Specific tool used* |  |  | *Baseline/Included in outcomes analysis* | *Primary Outcomes* |  |
| **Bischoff et al., 2012**  **The Netherlands** | Advanced Practice Nurse (APN) - Case Manager | Y | COPD Self-Efficacy Scale (CSES) | The self-management program consisted of paper modules and a written exacerbation action plan. The practice nurse applied the program to the individual patient in two to four sessions of approximately one hour each, scheduled in four to six consecutive weeks, followed by telephone calls/General practice. | COPD | 110/110 | Quality of Life | High |
| **Bosworth et al., 2005**  **USA** | Advanced Practice Nurse (APN) - Case Manager | Y | 1) Measure of self-confidence with ipertension treatment Scale 2) Morisky Self-reported medication-taking Scale | Telephone contacts every 2 months for 24 months. The nurse delivers both tailored and standard information in nine modules/Primary care clinic. | Hypertension | 588/588 | Primary outcome not evaluated (Only secondary outcome reported) | Moderate |
| **Bosworth et al., 2009**  **USA** | Registered Nurse (RN) | N |  | See Bosworth 2005 | Hypertension | 319/252 (Only UC and Behavioral Intervention at 24 months) | BP control | High |
| **Boyd et al., 2010**  **USA** | Registered Nurse (RN) | Y | Patient Assessment of Chronic Illness Care (PACIC) | A comprehensive assessment at home, creation and maintenance of an evidence based "Care Guide" (care plan) and an Action Plane (patient's self-care plane), monthly monitoring, coaching for self-management, smoothing transition into and out of hospitals, coordinating all providers of care, educating and supporting family caregivers and accessing community resources/Patient’s home | Multichronic | 904/846 | Patient Assessment of Chronic Illness Care (Goal setting; Coordination of care; Decision support; Problem solving; Patient activation; Aggregate quality) | Low |
| **Cooper et al., 2008**  **UK** | Advanced Practice Nurse (APN) - Nurse Specialist (NS) | Y |  | Health educational program – LAY (Look After Yourself) for physical activities and exercise, relaxation, health topics. 2 hours sessions weekly for 8 weeks/Hospital diabetes outpatient clinics and General Practice center | Diabetes | 59/59 (Only short term follow-up, 6 months) | HbA1c | Moderate |
| **Delaney et al., 2008**  **UK** | Registered Nurse (RN) | N |  | Attendance of secondary prevention nurse-led clinics during which patients’ symptoms and treatment were reviewed, use of aspirin promoted, blood pressure and lipid management reviewed, lifestyle factors assessed and, if appropriate, behavioral changes negotiated/Secondary prevention nurse-led clinics in general practice | Coronary heart disease | 1343/1318 | Total Mortality; Coronary events | Moderate |
| **Denver et al., 2003**  **USA** | Registered Nurse (RN) | N |  | The hypertension nurse emphasized the need for tight BP control, gave non pharmacological advice for healthy living, and (if necessary) discussed problems regarding side effects of existing antihypertensive treatment/Outpatient nurse-led clinics from the hospital diabetes clinic | Multichronic | 120/115 | SBP, DBP | Low |
| **Gabbay et al., 2013**  **USA** | Registered Nurse (RN) - BSN level | Y | Summary of Diabetes Self-care Activities (SDSCA) | The intervention group met individually within their primary care clinic with their nurse case managers at baseline, 2 weeks, 6 weeks, 3 months, 6 months, 12 months, and at least every 6 months thereafter. Visits were approximately 1-hour long. Participants intervention group could also contact their NCM (nurse case manager) by phone calls or e-mails between visits when appropriate/Primary care clinic | Diabetes | 545/421 | HbA1c; LDL; SBP; DBP; Diabetes-related emotional distress; Satisfaction with the diabetes regimen ; Impact of diabetes on quality of life; Depression symptoms; Self-care activities | Low |
| **Galbreath et al., 2004**  **USA** | Advanced Practice Nurse (APN)- Disease Manager | Y |  | Telephone education. In the event that a patient was thought to be unstable by the disease manager, face-to-face evaluation with a home healthcare nurse could be arranged. Initial call frequency was weekly, with a transition to monthly/Patient’s home | Chronic heart failure | 1069/1069 | Total mortality | Low |
| **Garcia-Peña et al., 2001**  **Mexico** | Registered Nurse (RN) | Y |  | Regular home visits from a nurse over 6 months with blood pressure measurement, information from the baseline health check, discussion about possible healthier lifestyle changes, suggestion of different alternative ways to achieve the changes with negotiation of specific target. Review of the pharmacological treatment and adherence encouragement/Patient’s home | Hypertension | 718/683 | Reduction in SBP; Reduction in DPB | Moderate |
| **Gary et al., 2003**  **USA** | Advanced Practice Nurse (APN) | N |  | Home telephonic calls. The model incorporates critical constructs from adult learning, social support, and behavior modification theories and health services research such as predisposing, reinforcing, and enabling factors/Patient’s home | Diabetes | NA/72 (only UC and NCM groups) | HbA1c | Low |
| **Goudswaard et al., 2004**  **The Netherlands** | Registered Nurse (RN) | N |  | One-to-one sessions focused on: general information on diabetes (monitoring home blood pressure and home glucose levels); reinforcing compliance with actual medication; importance of physical exercise and losing body weight; and nutritional advice. During the 6-month period, six sessions were given, at intervals of 3–6 weeks/General practice | Diabetes | 58/54 | HbA1c | High |
| **Ishani et al., 2011**  **USA** | Advanced Practice Nurse (APN) - Infermiere Case Manager | N |  | Patients, in collaboration with the study nurses, established lifestyle modification goals and developed personal action plans. Contacts every 2 weeks initially and for the frequency of contact to decrease as the patient achieved home BP and glucose goals. The study duration was 12 months/Patient’s home | Diabetes | 556/556* | % achieving BP 130/80mmHg; LDL 100mg/dL; HbA1c ,8.0% | High |
| **Krein et al., 2004**  **USA** | Advanced Practice Nurse (APN) - Nurse Practitioner Case Manager | Y |  | Patient contact occurred primarily by telephone, although face-to-face visits could be arranged. Case managers were directed to encourage patient self-management, including diet and exercise; provide reminders for recommended screenings/tests; help with appointment scheduling; monitor home glucose and home blood pressure levels; and identify and initiate medication and dose changes as needed/Outpatient case management | Diabetes | 246/209 | HbA1c ; LDL ; SBP ; DBP | Moderate |
| **Lee et al., 2007**  **UK** | Nurse Specialist (NS) - Public Health Nurse | N |  | Six-month community-based walking intervention delivered by the public health nurse. A series of regular individual contacts was provided through telephone and face-to-face visits/Local community activity centers and patient’s home | Hypertension | 202/184 | Change in SBP; Reduction in DBP | High |
| **Murchie et al., 2003**  **UK** | Registered Nurse (RN) | N |  | See Delaney et al., 2008 | Coronary heart disease | 1343/1098 | Use of secondary prevention (aspirin , BP managemet , lipid management , healthy diet, exercise , non-smoking); Total Mortality; Coronary events | Moderate |
| **Murchie et al., 2004**  **UK** | Registered Nurse (RN) | N |  | See Delaney et al., 2008 | Coronary heart disease | 1343/961 | Quality of Life; Anxiety; Depression; Chest pain; Worsening chest pain | Low |
| **Piette et al., 2000**  **USA** | Registered Nurse (RN) | N |  | Automated telephone calls were used to deliver targeted and tailored self-care education messages/General medicine clinic | Diabetes | 280/248* | Glucose self-monitoring; Foot inspection self-monitoring; Weight self-monitoring; Perceived glycemic control; Diabetes-related symptoms ; HbA1c; Serum Glucose | Low |
| **Rudd et al., 2004**  **USA** | Advanced Practice Nurse (APN) - Case Manager | Y |  | Nurse counseling at baseline on correct use of the automated home BP device, regular return of the automatically printed BP reports, tips for enhancing drug adherence, and recognition of potential drug side effects. Follow up phone contacts at 1 week and at 1, 2, and 4 months/Patient’s home | Hypertension | 150/137 | Reduction in DBP; Reduction in SBP; Medication adherence; Antihypertensive medications changes | High |
| **Shea et al., 2006**  **USA** | Advanced Practice Nurse (APN) - Nurse Case Manager | Y |  | Home telemedicine unit (HTU). Nurse case managers were trained in diabetes management and in the use of computer-based case management tools to facilitate interactions through videoconferencing with patients/Patient’s home | Diabetes | 1665/1657* | HbA1c; SBP; DBP ; LDL; Total Cholesterol | High |
| **Shea et al., 2009**  **USA** | Advanced Practice Nurse (APN) - Nurse Case Manager | Y |  | See Shea et al., 2006 | Diabetes | 1665/1663* | HbA1c ; SBP ; DBP; LDL | Moderate |
| **Sisk et al., 2006**  **USA** | Advanced Practice Nurse (APN) - Case Manager | Y |  | Face-to-face visit at baseline, home telephone follow-up/Community hospitals | Chronic heart failure | 406/406 | Hospitalizations; Functioning (physical component); Mortality | High |
| **Taylor et al., 2003**  **USA** | Advanced Practice Nurse (APN) Care Manager - Diabetes Nurse-Care Manager | Y |  | All intervention patients were asked to attend a 1- to 2-h group class that met once a week for 4 weeks. Telephone follow-up calls/Primary care center and patient’s home | Multichronic | 169/127 | HbA1c ; Total cholesterol; LDL ; HDL; Triglycerides; Glucose; SBP; DBP; BMI; dilated eye exam; Flu shot; Foot exam; Dental exam; Quality of life; Depression; Patients satisfaction; Physician satisfaction; physician’s visits; Hospitalization; Emergency room | Low |
| **ter Bogt et al., 2009**  **The Netherlands** | Advanced Practice Nurse (APN) - Nurse Practitioner | Y |  | Four individual visits and one feedback session by telephone in the first year/Patient’s home | Multichronic | 457/416 | Outcomes evaluated in subgroups of women and men: Weight ; Weight % ; Waist ; SBP ; DBP; Total cholesterol; HDL ; LDL; Fasting glucose ; Weight losers and stabilizers | High |
| **ter Bogt et al., 2011**  **The Netherlands** | Advanced Practice Nurse (APN) - Nurse Practitioner | Y |  | See ter Bogt et al., 2009 | Multichronic | 457/357 | Weight ; Weight %; BMI; Waist ; SBP ; DBP; Total cholesterol; HDL ; LDL; Fasting glucose ; Impaired fasting glucose; Weight losers and stabilizers; Weight regainers | Moderate |
| **Tonstad et al., 2007**  **Norway** | Registered Nurse (RN) | N |  | Monthly meetings with the nurse for 6 months. The initial session lasted for 60 min and subsequent sessions lasted for 30 min/Patient’s home | Hypertension | 51/47 | Reduction in DBP; Reduction in SBP; Number of Metabolic syndrome risk factors (glucose, Hb1Ac, triglyceride concentrantions, total cholesterol , waist circumference, weight ) | Low |
| **Walters et al., 2013**  **Australia** | Registered Nurse (RN) - Community Health Nurse | Y | Self Efficacy for managing Chronic Disease (SEMCD) | Telephone calls 16×30 min over 12 months, with increasing time between calls/Patient’s home | COPD | 182/154 | Quality of life | Moderate |
| **Woollard et al., 2003a**  **Australia** | Registered Nurse (RN) | Y |  | The high level intervention group were counselled in individual sessions up to 60 min every month over a period of 12 months. Participants were provided with a personalized educational manual developed to support the cognitive behavioral approach/General practice | Multichronic | 212/101 | Total Energy intake; Total Fat; Satured Fat; Polyunsatured Fat; Monousatured Fat; Sodium; Potassium; Fibre; Alcohol; Total cholesterol; LDL; HDL; triglycerides; n3/n6 fatty acids; BMI; Weight; Waist to hip ratio | Low |
| **Woollard et al., 2003b**  **Australia** | Registered Nurse (RN) | Y |  | See Wollard et al., 2003a | Multichronic | 212/71 | SBP ; DBP ; 24h SBP ; 24h DBP; Awake SBP ; Awake DBP; Asleep SBP; Asleep DBP; 24h Heart rate ; BMI; Weight; Energy intake; Fibre Intake; Alcohol Intake; Physical activity; Fasting blood sugar; glycated haemoglobin; Urinary sodium; Urinary Potassium | Low |
