# Supplementary material for: Are community-based nurse-led self-management support interventions effective in chronic patients? Results of a systematic review and meta-analysis
Source: PLoS One. 2017 Mar 10;12(3):e0173617. doi: 10.1371/journal.pone.0173617 (PMC5345844; doi:10.1371/journal.pone.0173617)
Supplement: S2 Table — (DOCX) [file pone.0173617.s004.docx]

| **TITLE** | **SEQUENCE GENERATION** | **ALLOCATION CONCEALMENT** | **BLINDING** | **INCOMPLETE OUTCOME DATA** | **SELECTIVE OUTCOME REPORTING** | **OTHER SOURCES OF BIAS** | **OVERALL QUALITY**  **SCORE** |
| --- | --- | --- | --- | --- | --- | --- | --- |
| Garcia-Peña 2001/Mexico | Low risk | Uncertain risk | Low risk | Low risk | Low risk | Uncertain risk | ***Moderate (2)*** |
| Murchie 2003/UK | Low risk | Uncertain risk | Uncertain risk | Low risk | Low risk | Uncertain risk | ***Moderate (3)*** |
| Murchie 2004/UK | Low risk | Uncertain risk | High risk | Low risk | Low risk | Uncertain risk | ***Low (4)*** |
| Galbreath 2004/USA | Uncertain risk | Uncertain risk | Uncertain risk | Low risk | Low risk | Uncertain risk | ***Low (4)*** |
| Rudd 2004/USA | Low risk | Uncertain risk | Low risk | Low risk | Low risk | Low risk | ***High (1)*** |
| Bosworth  2005/USA | Low risk | Low risk | High risk | Low risk | Low risk | Uncertain risk | ***Moderate (3)*** |
| Sisk  2006/USA | Low risk | Low risk | Uncertain risk | Low risk | Low risk | Low risk | ***High (1)*** |
| Tondstad  2006/Norway | Low risk | Low risk | High risk | Low risk | Low risk | High risk | ***Low (4)*** |
| Lee 2007/UK | Low risk | Low risk | Low risk | Low risk | Low risk | Uncertain risk | ***High (1)*** |
| Delaney 2008/UK | Low risk | Uncertain risk | Uncertain risk | Low risk | Low risk | Uncertain risk | ***Moderate (3)*** |
| Bosworth 2009/USA | Low risk | Low risk | Low risk | Low risk | Low risk | Uncertain risk | ***High (1)*** |
| Bischoff 2012/  The Netherlands | Low risk | Uncertain risk | Low risk | Low risk | Low risk | Low risk | ***High (1)*** |
| Walters 2013/Australia | Low risk | Low risk | Low risk | High risk | Low risk | Uncertain risk | ***Moderate (3)*** |
| Cooper 2008/UK | Uncertain risk | Uncertain risk | Low risk | Low risk | Low risk | Low risk | ***Moderate (2)*** |
| Gabbay 2013/USA | Uncertain risk | Uncertain risk | High risk | High risk | High risk | Low risk | ***Low (8)*** |
| Gary 2003/USA | Low risk | Uncertain risk | Low risk | High risk | Low risk | Uncertain risk | ***Low (4)*** |
| Goudswaard 2003/  The Netherlands | Low risk | Low risk | Low risk | Low risk | Low risk | Low risk | ***High (0)*** |
| Ishani 2011/USA | Low risk | Uncertain risk | Low risk | Low risk | Low risk | Low risk | ***High (1)*** |
| Krein 2004/USA | Uncertain risk | Uncertain risk | Low risk | Low risk | Low risk | Low risk | ***Moderate (2)*** |
| Piette 2000/USA | Low risk | Uncertain risk | High risk | Low risk | Low risk | Uncertain risk | ***Low (4)*** |
| Shea 2006/USA | Low risk | Low risk | Low risk | Low risk | Low risk | Uncertain risk | ***High (1)*** |
| Shea 2009/USA | Low risk | Low risk | Low risk | High risk | Low risk | Low risk | ***Moderate (2)*** |
| Boyd 2009/USA | Uncertain risk | Low risk | High risk | Uncertain risk | Low risk | High risk | ***Low (6)*** |
| Denver 2003/USA | Uncertain risk | High risk | Low risk | Low risk | Low risk | Uncertain risk | ***Low (4)*** |
| Taylor 2003/USA | Uncertain risk | Uncertain risk | High risk | High risk | High risk | Uncertain risk | ***Low (9)*** |
| Ter Bogt 2009/ The Netherlands | Low risk | Uncertain risk | Low risk | Low risk | Low risk | Low risk | ***High (1)*** |
| Ter Bogt 2011/ The Netherlands | Low risk | Uncertain risk | Low risk | High risk | Low risk | Low risk | ***Moderate (3)*** |
| Wollard 2003/ Australia | Uncertain risk | Uncertain risk | Low risk | High risk | Low risk | Uncertain risk | ***Low (5)*** |
| Wollard 2003b/ Australia | Uncertain risk | Uncertain risk | Low risk | High risk | Low risk | Uncertain risk | ***Low (5)*** |
| A quantitative score was assigned to each judgement as follows: Low risk of bias=0; Uncertain risk of bias=1; High risk of bias=2. An overall quality score was calculated by summing the values of the different items using the following scale: overall score ≤1 High Quality; overall score ≤3 Moderate Quality; overall score >3 Low Quality. | | | | | | | |
